# Umbilical Cord Blood Transplantation in Lesch-Nyhan Syndrome: A Case Report and Literature Review

**DOI:** 10.7759/cureus.97008

**Published:** 2025-11-16

**Authors:** Te-Fu Weng, Ju-Pi Li, Chung-Han Tin, Kang-Hsi Wu

**Affiliations:** 1 Department of Pediatrics, Chung Shan Medical University Hospital, Taichung, TWN; 2 Department of Pathology, School of Medicine, Chung Shan Medical University, Taichung, TWN; 3 Pediatrics, Chang Gung University School of Business, Taipei, TWN

**Keywords:** hematopoietic stem cell transplantation, hprt deficiency, inborn errors of metabolism, lesch-nyhan syndrome, umbilical cord blood transplantation

## Abstract

Lesch-Nyhan syndrome (LNS) is a rare X-linked disorder caused by hypoxanthine phosphoribosyltransferase 1 (HPRT1) gene mutations, leading to HPRT1 deficiency, hyperuricemia, and severe neurological dysfunction, including self-injurious behavior. Hematopoietic stem cell transplantation (HSCT) has been applied to some inborn errors of metabolism (IEM), yet only a few cases with limited success in LNS have been reported. This case was diagnosed with LNS at nine months of age due to developmental delays. He subsequently received 5/6 human leukocyte antigen-matched umbilical cord blood transplantation (UCBT) at 14 months of age. Myeloablative conditioning included fludarabine, busulfan, and antithymocyte globulin. This patient achieved complete donor chimerism on day 32 after UCBT. Before UCBT, the patient’s HPRT1 protein was lower than his parents, but the levels increased after UCBT. At 36 months of age, no self-mutilation was noted, and neurological improvement was found. HSCT is more effective in preventing disease progression in IEM than in reversing established manifestations, making early diagnosis critical. In this case, LNS was diagnosed early, allowing prompt HSCT. To our knowledge, this is the youngest LNS patient to receive HSCT. We propose that early HSCT may contribute to neurological improvements. Early UCBT is feasible and may prevent self-mutilation and promote neurological recovery in LNS. Further studies are needed.

## Introduction

Lesch-Nyhan syndrome (LNS) is a rare X-linked recessive disorder caused by mutations in the hypoxanthine phosphoribosyltransferase 1 (HPRT1) gene, resulting in HPRT1 deficiency [1]. This enzyme is essential for purine salvage, and its absence leads to hyperuricemia, gouty arthritis, and severe neurological symptoms, including intellectual disability, dystonia, and self-mutilation, which typically manifest by age 2 [1]. Allopurinol controls hyperuricemia but does not prevent neurological deterioration. Unlike lysosomal storage disorders that can benefit from enzyme replacement therapy [2], LNS is a purine metabolism defect for which no enzyme replacement option exists; therefore, alternative therapeutic approaches such as hematopoietic stem cell transplantation (HSCT) are being explored.

HSCT has been proposed to provide missing enzymes in some inborn errors of metabolism (IEM) and may cross the blood-brain barrier to ameliorate neurological deficits [3,4]. In addition, in certain IEMs, donor-derived hematopoietic stem cells can differentiate into microglia-like cells within the central nervous system (CNS), delivering functional enzyme locally [3-5]. Early HSCT has preserved neurodevelopmental potential in these disorders. Therefore, we propose that early HSCT may prevent CNS damage in LNS. HSCT has been proposed to provide missing enzymes in some IEM and may cross the blood-brain barrier to ameliorate neurological deficits [3,4]. Additionally, in some IEMs, donor hematopoietic stem cells can differentiate into microglia-like cells in the central nervous system (CNS), delivering functional enzyme locally [3-5]. Early HSCT has preserved neurodevelopmental potential in these disorders. Therefore, we propose that early HSCT may prevent CNS damage in LNS.

Here, we report the youngest known case of umbilical cord blood transplantation (UCBT) in a 14-month-old boy with LNS. We review previous LNS cases treated with HSCT and discuss new strategies such as induced pluripotent stem cells (iPSCs) and gene therapy.

## Case presentation

A six-month-old boy presented with developmental delay, including hypotonia, inability to roll over, and intermittent opisthotonus. Between six and nine months of age, the patient was unable to lift his head, roll over, or produce purposeful vocalizations, and social smiling markedly decreased. He exhibited generalized hypotonia, intermittent opisthotonus, poor response to people, and a short attention span. He was born full term without perinatal asphyxia or infection, and there was no family history of similar disorders or early male deaths. At the time of diagnosis, self-injurious behaviors such as lip or hand biting had not yet developed. Biochemical studies revealed hyperuricemia (serum uric acid 10.8 mg/dL) with an increased urinary uric acid/creatinine ratio, while other hematologic and biochemical tests were normal. HPRT enzyme activity was not assessed because of technical limitations. Brain MRI demonstrated diffuse cerebral atrophy, and EEG revealed left posterior epileptiform discharges corresponding to right focal motor seizures. These findings led to the suspicion of a purine metabolism disorder, and subsequent HPRT1 gene analysis confirmed LNS via genetic examination (HPRT1 NM_032578.4:c.880delG, hemizygous), and his mother was confirmed as a carrier. Hyperuricemia (initial uric acid 10.8 mg/dL) was managed with allopurinol, reducing levels to 8.0 mg/dL at 11 months. Due to progressive neurological deterioration and the absence of a matched sibling donor, UCBT was considered for early metabolic correction and neuroprotection. The umbilical cord blood donor was genetically unaffected, with no pathogenic variant detected in HPRT1.

At age 14 months, myeloablative conditioning was administered according to the European Society for Blood and Marrow Transplantation (EBMT)/European Society for Immunodeficiencies (ESID) Protocol A for unrelated UCBT [6]. The regimen included antithymocyte globulin (2.5 mg/kg/day, days -9 to -6), fludarabine (40 mg/m²/day, days -5 to -2), and busulfan (5.1 mg/kg/day in 4 doses, days -5 to -2), with levetiracetam (10 mg/kg/dose twice daily) for seizure prophylaxis. After conditioning, one unit of 5/6 human leukocyte antigen (HLA)-matched unrelated UCB (10.8 × 10⁷ total nucleated cells/kg, 4.9 × 10⁵ CD34⁺/kg) was infused. Graft-versus-host disease (GvHD) prophylaxis included cyclosporine A (target trough level: 200-300 ng/ml) and mycophenolate mofetil (15 mg/kg/dose twice daily) from day 1 after UCBT.

Ursodeoxycholic acid and enoxaparin were administered for veno-occlusive disease prevention. Prophylactic antibiotics included ciprofloxacin, micafungin, trimethoprim-sulfamethoxazole, and acyclovir. Engraftment was achieved (absolute neutrophil count >1,500/μL on day 21; platelets >50,000/μL on day 32). Grade II acute GvHD on day 25 resolved with methylprednisolone treatment; no severe infection occurred. The patient achieved complete donor chimerism on day 32.

**Figure 1:**
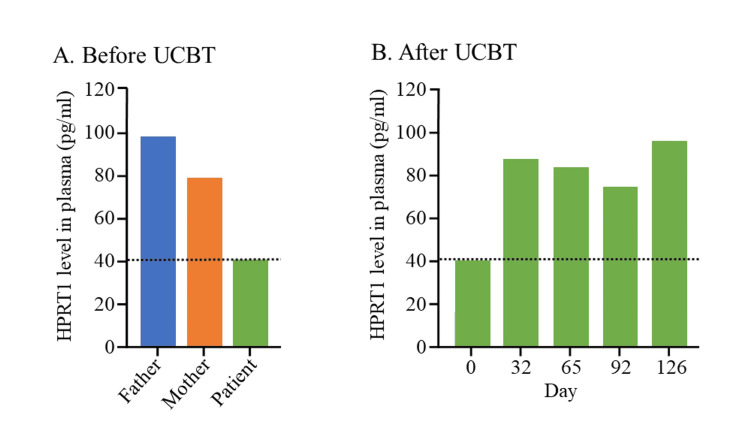
Hypoxanthine phosphoribosyltransferase 1 (HPRT1) protein levels HPRT1 protein levels in our patient's parents and our patient before and after umbilical cord blood transplantation (UCBT): (A) lower HPRT1 protein level in our patient before UCBT than those of his parents; (B) HPRTI protein levels in our patient increased after UCBT.

HPRT1 protein was measured by enzyme-linked immunosorbent assay (MBS2019477, MyBioSource, Inc.). Before UCBT, the patient’s HPRT1 protein was 40.9 pg/ml, which was significantly lower than his parents (father: 98.8 pg/ml; mother: 78.9 pg/ml) (Figure 1A). After UCBT, the HPRT1 protein increased to 191.1 pg/ml on day 32, 82.5 pg/ml on day 65, 75.3 pg/ml on day 92, 97.0 pg/ml on day 126, respectively (Figure 1B). These findings documented that the HPRT1 protein in this patient was low before UCBT. However, the HPRT1 protein increased after UCBT and reached the levels of his patients. Neurological improvements included reduced opisthotonus, decreased dystonia/spasticity, improved facial expressions, increased social interaction, and developmental progress. No self-injurious behavior, such as lip biting, was noted at 36 months of age.

## Discussion

The feasibility of allogeneic HSCT in LNS is supported by its proven efficacy in other IEMs, such as Hurler [7-9] and Krabbe disease [10-14], where donor-derived cells supplement deficient enzyme or promote repair. Compared with other allogeneic HSCT sources, umbilical cord blood offers advantages including rapid availability, better HLA mismatch tolerance, and lower risk of GvHD [15]. Because IEM patients are young and have low body weight, the cell dose in umbilical cord blood is generally sufficient; thus, UCBT is a feasible option for LNS.

Previous reports illustrate both the challenges and potential of HSCT for LNS. Endres et al. (1991) described early post-transplant death at 16 months of age [16], highlighting the need for improved protocols, while Kállay et al. (2012) reported neurological improvement and absence of self-injury in a two-year-old after UCBT [17]. In our patient, UCBT performed according to the EBMT/ESID protocol achieved full donor chimerism, increased HPRT1 protein levels, and clinical neurological improvement without severe complications. At the time of submission (10 months post-UCBT, July 2025), he remained free of chronic GvHD or severe infection. He had mild acute gastrointestinal GvHD (grades I-II), which resolved with corticosteroids, and continued on cyclosporine prophylaxis complicated by chronic hyperkalemia. Longer follow-up is ongoing to monitor for late transplant-related events. A comparison of reported and current cases is summarized in Table 1.

**Table 1:** Comparison of hematopoietic stem cell transplantation in previously reported Lesch-Nyhan syndrome cases and our patient

Study	Age at transplantation	Donor	Conditioning	Outcome
Endres et al. (1991) [16]	16 months	Bone marrow	Busulfan/cyclophosphamide	Death (day +10), no engraftment
Kállay et al. (2012) [17]	24 months	Umbilical cord blood (6/6 human leukocyte antigen)	Busulfan/cyclophosphamide/antithymocyte globulin	Full chimerism, no self-injury
This case (2025)	14 months	Umbilical cord blood (5/6 human leukocyte antigen)	Busulfan/fludarabine/antithymocyte globulin	Full chimerism, no self-injury, neurologic improvement

Although formal criteria for HSCT in LNS have not been established, decision-making can be guided by principles from other metabolic disorders where early transplantation preserves neurological potential [3,4]. HSCT may be considered in genetically confirmed HPRT1 deficiency diagnosed before irreversible neurological injury (typically <2 years of age), showing progressive neurodevelopmental delay despite standard therapy, and with an appropriate HLA-matched or suitably matched umbilical cord blood donor. Multidisciplinary evaluation is essential to balance potential benefit and risk.

The ultimate objective of HSCT in LNS is not to reverse established neurological injury but to achieve neuroprotection and metabolic stabilization through donor-derived restoration of systemic and CNS purine metabolism. Early transplantation may allow donor cells to engraft and differentiate into microglia-like cells that provide functional HPRT enzyme before irreversible neuronal loss.

In this patient, HPRT1 protein levels, initially lower than those of both parents, increased after UCBT to comparable levels, suggesting metabolic correction from donor-derived cells. Correspondingly, neurological function improved, possibly reflecting both peripheral and CNS-level enzyme restoration. Interpretation of HPRT1 protein levels should consider possible inter-assay variability and the influence of concurrent medications or supportive therapies.

Although neurological and developmental improvements were evident from multidisciplinary evaluations, standardized neurodevelopmental assessment tools were not used due to the patient’s young age and disease stage. Future studies should incorporate objective scales such as the Bayley Scales or Gross Motor Function Measure (GMFM) to better quantify post-transplant neurodevelopmental outcomes.

The implementation of UCBT in LNS also raises ethical and logistical considerations. As the procedure remains experimental with uncertain long-term benefit, decisions must follow ethical review and transparent family counseling. In our case, the decision was reached through multidisciplinary consensus involving pediatric hematology, neurology, metabolic, rehabilitation, and ethics specialists, with informed parental consent. Broader application of UCBT requires centers with adequate expertise, infrastructure, and long-term neurodevelopmental follow-up. In resource-limited settings, these requirements may restrict feasibility and increase procedural risks. Until more evidence becomes available, UCBT for LNS should be performed only in specialized centers with multidisciplinary coordination and ethical oversight.

Emerging therapies for LNS include gene therapy and iPSC-based approaches [18]. Gene therapy using lentiviral or CRISPR/Cas9-mediated editing aims to correct HPRT1 mutations in hematopoietic or neural cells, potentially offering a less invasive alternative to allogeneic HSCT [19]. iPSC-derived neuronal models also provide platforms for drug discovery to rescue LNS-associated phenotypes [20].

## Conclusions

With advances in molecular diagnosis, some IEMs can be diagnosed early and treated timely. As time goes by, LNS will result in irreversible neurological damage. Therefore, we speculate that early HSCT in LNS preserves neurodevelopmental potential similar to Hurler syndrome and Krabbe disease. This case is the youngest LNS patient receiving HSCT. The early UCBT may be associated with neurological improvement. Further studies are warranted.

Objective standardized neurodevelopmental assessments were not applied in this single case, which limits the strength of conclusions regarding neurological recovery. As a case report, these observations are limited by the absence of controls and may not be generalizable.
